# Impact of Horn Traits on Yanhuang Cattle: Association with Production Performance and Genetic Characterization of Candidate Polled Alleles

**DOI:** 10.3390/ani16081179

**Published:** 2026-04-12

**Authors:** Shengxue Sima, Zewen Wu, Xinxin Zhang, Guangyao Meng, Tianqi Si, Wenyu Jiao, Ziqi Liu, Tianyu Zhang, Yunkai He, Guangjun Xia

**Affiliations:** 1 Agriculture College, Yanbian University, Yanji 133002, China; shengxue202308@163.com (S.S.);; 2 Heilongjiang Province Endangered Wildlife Rescue and Breeding Center, Harbin 150090, China; 3 Engineering Research Center of North-East Cold Region Beef Cattle Science & Technology Innovation, Ministry of Education, Yanbian University, Yanji 133002, China

**Keywords:** Yanhuang cattle, polled trait, molecular identification, growth performance

## Abstract

Yanhuang cattle is a specialized beef breed independently developed in China with high economic value. However, the effects of the horned/polled trait on its production performance and the underlying genetic markers are poorly understood. This study aimed to evaluate the impact of horn status and to validate candidate genetic markers for the polled trait in this breed. A total of 60 Yanhuang cattle (30 horned and 30 polled) were used for comparative analysis of growth, carcass, and meat quality traits. Additionally, blood samples from 200 individuals (100 horned and 100 polled) were collected for molecular screening of seven candidate genetic markers previously associated with polledness in cattle. The polled cattle showed significantly higher body weight from 12 months onward. Body height and hip height were significantly greater at 12, 18, and 24 months, while body length displayed significant advantages at 6, 18, 24, and 30 months. Polled cattle also outperformed in chest girth (18 months), abdominal circumference (significant at 18–30 months), and ischial end width (significant at 6–12 months). Regarding slaughter traits, polled cattle exhibited higher carcass weight, along with significantly improved slaughter and net meat rates, though no differences were observed in meat quality. At the molecular level, the P_202ID_ indel marker was successfully validated in the polled Yanhuang population. Our findings demonstrate that the polled trait is associated with superior production performance in Yanhuang cattle, and the P_202ID_ marker represents a reliable molecular tool for polled selection, providing a theoretical and technical basis for the genetic improvement and breeding of this breed.

## Simple Summary

Yanhuang cattle is a specialized beef breed independently developed in China, possessing significant economic value. Currently, the influence of horn traits on its growth performance and the applicability of known molecular markers for polledness in this breed remain unclear. In this study, we compared the production performance of 30 polled and 30 horned Yanhuang cattle. Concurrently, candidate genetic markers for the polled trait reported in other breeds were screened in 200 individuals (100 polled and 100 horned) using molecular methods. Our results showed that polled Yanhuang cattle exhibited superior growth and carcass traits at various developmental stages. Notably, the candidate marker P_202ID_ was identified and validated in the polled population, demonstrating its potential utility in marker-assisted selection for Yanhuang cattle breeding programs.

## 1. Introduction

Horn characteristics are important biological traits of ruminants and exert multifaceted impacts on animal husbandry production, involving core issues such as animal welfare, management costs, and safety [1]. With the intensification of livestock production, the presence or absence of horns has become a key factor in breeding and management decisions. Polled herds exhibit a significantly higher incidence of injuries caused by agonistic interactions, which increases management difficulty and costs [2]. Therefore, eliminating horns through either dehorning techniques or the selection and breeding of polled breeds has gained increasing attention worldwide. From an economic perspective, the selection and breeding of polled cattle are generally more cost-effective than traditional physical or chemical dehorning in intensive rearing systems [3].

The horned/polled trait in cattle has been confirmed to be an autosomal dominant inheritance trait [4]. The candidate locus for the polled phenotype in cattle is mapped to the centromeric region of bovine chromosome 1 (BTA1) [5]. In recent years, with the advancement of molecular biology techniques, the genetic basis of this region has been thoroughly elucidated, and multiple candidate genetic markers co-segregating with the polled phenotype have been identified, laying a foundation for understanding its molecular mechanism and conducting molecular breeding. Among numerous cattle breeds, breed-specific genetic loci have been discovered by researchers. Among them, the P_202ID_ mutation was first identified in European beef cattle breeds; this variant is a complex indel, in which a 212 bp sequence (1,705,834–1,706,045 bp) on chromosome 1 is duplicated and replaces the original 10 bp sequence (1,706,051–1,706,060 bp), resulting in an insertion of 202 bp [6]. Studies have confirmed that individuals carrying this mutation exhibit the polled phenotype. Another study on dairy breeds such as Holstein cattle revealed a 260 kb haplotype containing multiple tightly linked mutation sites [7]. This haplotype contains at least five candidate mutations, including three single nucleotide polymorphisms (SNPs): P_G1855898A_ (G > A at position 1,855,898), P_G1654405A_ (G > A at position 1,654,405), and P_C1768587A_ (C > A at position 1,768,587), as well as two structural variants: P_80kbID_ (an 80 kb DNA fragment duplication accompanied by a 2 bp deletion) and P_5ID_ (a 12 bp sequence “ttctcagaatag” replaces the 7 bp sequence “cgcatca”, leading to an insertion of 5 bp). In addition, a novel mutation, P_219ID_, was discovered in Mongolian yaks, characterized by a 219 bp duplicated insertion at 61 bp downstream of the original sequence (starting at position 1,976,128 bp) [8]. These established molecular markers provide important genetic tools and targets for the efficient and accurate marker-assisted selection of the polled trait in different cattle breeds.

Yanhuang Cattle is an indigenous specialized beef cattle breed independently developed in China and nationally approved in 2008. It was developed by crossbreeding Limousin bulls (sire) with Yanbian Yellow Cattle cows (dam), and exhibits excellent production performance and adaptability [9]. However, no systematic studies have been conducted to investigate the effects of the horned/polled trait on the growth, development, and production performance of Yanhuang Cattle. Moreover, the applicability and genetic characteristics of the aforementioned known candidate molecular markers in this breed remain entirely unknown. Therefore, to fill the research gap in this field and provide a theoretical basis for breeding new polled lines of Yanhuang Cattle, this study aims to systematically compare the differences in growth, carcass, and meat quality traits between horned and polled Yanhuang Cattle to evaluate the impact of the horn trait on production performance. Meanwhile, molecular biological methods will be used to verify the applicability of multiple known candidate genetic markers (e.g., P_202ID_, P_219ID_) in the Yanhuang Cattle population, thereby providing reliable tools for the marker-assisted selection of new polled lines.

## 2. Materials and Methods

All experimental procedures in this study were conducted in strict accordance with the guidelines established by the Regulations on the Management of Laboratory Animals (Ministry of Science and Technology, China, 2017) and were approved by the Medical Ethics Committee of Yanbian University (Approval No.: 201702).

### 2.1. Experimental Design and Animal Management

Sixty 6-month-old Yanhuang cattle bulls (30 polled and 30 horned), all of which were healthy, were selected from Jixing Animal Husbandry Co., Ltd. in Hunchun City, Jilin Province, China. All these 60 bulls were healthy. Horn status was determined by visual inspection and manual palpation. Growth performance parameters were measured at 6, 12, 18, 24, and 30 months of age. All cattle were maintained under standardized feeding and management conditions until slaughter at 30 months of age. After slaughter, carcass weight, dressing percentage, and net meat percentage were measured, and meat quality evaluation was performed on Longissimus dorsi samples. For molecular analysis, blood samples were additionally collected from 200 Yanhuang cattle (100 polled and 100 horned) and stored at −20 °C.

### 2.2. Measurement of Production Performance Traits

Body weight (BW), body height (BH), hip height (HH), body length (BL), chest girth (CG), abdominal circumference (AC), and ischial end width (IEW) were measured at 6, 12, 18, 24, and 30 months of age. BW was measured using an electronic scale (SCS-3t; Tangshan Zhengxing Electronic Weighing Apparatus Co., Ltd., Tangshan, China) sensitivity ≤ 0.1 kg). Linear measurements (BH, HH, BL, CG, AC, IEW) were taken using a measuring stick (BMR-CZ; Beijing Beimuxin Trading Center, Beijing, China) and tape (DL270525; Ningbo Deli Tools Co., Ltd., Ningbo, China), and recorded in centimeters (cm).

At 30 months of age, the 60 experimental cattle were fasted for 18 h and then slaughtered by electric stunning followed by jugular exsanguination. After exsanguination and splitting, prior to washing, the hide, head, feet, tail, genital organs, surrounding fat and viscera were removed (with the kidneys and perirenal fat retained), and the carcass weight was recorded. The dressing percentage and net meat percentage were calculated as follows: Dressing percentage = (carcass weight/live weight) × 100%. Net meat percentage = (deboned meat weight/live weight) × 100%.

Meat quality was assessed on Longissimus dorsi (LD) muscle samples after 48 h of post-mortem storage at 4 °C. pH was measured using a portable pH meter (pH-STAR, MATTHAUS GmbH, Eckelsheim, Germany). Meat color (lightness L, redness a, yellowness b*) was determined using a colorimeter (OPTO-LAB, MATTHAUS GmbH, Eckelsheim, Germany). Cooking loss was determined as follows: approximately 100 g of longissimus dorsi muscle samples stored at 4 °C for 48 h were weighed after removal of surface fat and fascia, cooked in a water bath (HWS-26; Shanghai Yiheng Scientific Instrument Co., Ltd., Shanghai, China) until the core temperature reached 80 °C, and then cooled to room temperature. Surface moisture was absorbed with qualitative filter paper, and the samples were reweighed; cooking loss (%) was calculated using the formula: Cooking loss = (Pre-cooking weight—Post-cooking weight)/Pre-cooking weight × 100%. For shear force measurement, approximately 100 g of longissimus dorsi muscle samples stored at 4 °C for 48 h were heated in a constant-temperature water bath, with the core temperature monitored by a Mercury needle thermometer (WNG-01; Tianjin Tianke Glass Products Co., Ltd., Tianjin, China) until reaching 80 °C, followed by cooling to room temperature. Surface moisture was blotted with qualitative filter paper, and samples (6 cm × 3 cm × 3 cm) were cut parallel to the muscle fiber direction; shear force was then measured using a tenderness meter (C-LM3B; Beijing Tianxiang Feiyu Technology Co., Ltd., Beijing, China). Water-holding capacity (WHC) was assessed by the pressure method using a meat WHC analyzer (Model: RH-1000, Guangzhou Runhu Instrument Co., Ltd., Guangzhou China), with the instrument parameters set as a pressure weight of 25 kg and a compression time of 300 s. Meat samples were trimmed into cubes (approximately 2 cm × 2 cm × 2 cm) and weighed using an analytical balance (IS120AE; Ismart Biotechnology Ky, Espoo, Finland) to obtain the pre-compression weight (M1). Each sample was placed between 8 and 10 layers of filter paper on both sides and compressed on a support stand; after compression, the samples were removed, the adhered filter papers were discarded, and the samples were reweighed to obtain the post-compression weight (M2). WHC (%) was calculated using the formula: WHC = (M1 − M2)/M1 × 100%.

### 2.3. Detection of Candidate Genetic Markers

DNA samples were extracted from Yanhuang cattle blood following the manufacturer’s protocol of the TIANamp Blood DNA Kit (Tiangen Biotech, Beijing, China). The extracted DNA samples were analyzed using a UV spectrophotometer (NanoDrop One; Thermo Fisher Scientific, Waltham, MA, USA). The A260/A280 ratios were measured to verify sample purity, with acceptable values ranging between 1.8 and 2.0.

PCR primers were designed for seven candidate genetic markers (P_202ID_, P_80kbID_, P_5ID_, P_G1855898A_, P_C1768587A_, P_G1654405A_, and P_219ID_) associated with the polled phenotype in cattle [10]. The primer sequences and annealing temperatures for each PCR amplification are detailed in Table 1. In this study, the alleles associated with the polled trait in cattle were defined according to the standardized nomenclature system for livestock genetics. Pc denotes the Celtic polled allele carrying the P_202ID_ indel mutation, which harbors a 212 bp sequence duplication and a 10 bp segment replacement at positions 1,705,834–1,706,060 on chromosome 1. Prs refers to the wild-type allele without the P_202ID_ mutation, which is associated with the horned phenotype. Based on these definitions, the three genotypes are classified as follows: Pc/Pc represents the homozygous P_202ID_ mutation, Pc/Prs indicates the heterozygous P_202ID_ mutation, and Prs/Prs stands for the wild-type homozygote. Specifically, genotypes Pc/Pc and Pc/Prs correspond to the polled phenotype, while Prs/Prs corresponds to the horned phenotype.

The PCR reaction was performed in a 20 μL volume containing 10 μL of 2× Pfu PCR MasterMix (TIANGEN/KP201-02), 0.8 μM of each primer, approximately 50 ng of template DNA, and nuclease-free water. The thermal cycling program was: 95 °C for 5 min; 30 cycles of 94 °C for 30 s, annealing temperature for 30 s, 72 °C for 30 s; and a final extension at 72 °C for 10 min.

All PCR products and corresponding primers were aliquoted and sent to GENEWIZ (Tianjin, China) for sequencing analysis.

### 2.4. Data Analysis

Data on growth, carcass and meat quality traits were expressed as the mean ± standard deviation. Differences between the polled group and the horned group were analyzed using an independent samples *t*-test with SPSS 27.0 software (IBM Corp., Armonk, NY, USA). Statistical significance was defined as follows: *p* < 0.05 indicated a significant difference, *p* < 0.01 indicated a highly significant difference, and *p* > 0.05 indicated no significant difference. Genotypic frequencies were calculated using Microsoft Excel 2010.

## 3. Results

### 3.1. Growth Traits

At 6 months of age, polled cattle exhibited significantly greater BL (*p* < 0.05) and extremely significantly greater IEW (*p* < 0.01) compared to horned cattle. No significant differences were observed in BW, BH, HH, CG, or AC between the two groups (*p* > 0.05). By 12 months, polled cattle showed significantly greater BW, BH, and HH (*p* < 0.05), along with extremely significantly greater IEW (*p* < 0.01) than horned cattle. However, no significant differences were found in BL, CG, or AC (*p* > 0.05). At 18 months, polled cattle demonstrated extremely significant advantages in BW, BH, HH, and AC (*p* < 0.01), as well as significant advantages in BL and CG (*p* < 0.05) compared to horned cattle. IEW showed no significant difference between groups (*p* > 0.05). By 24 months, polled cattle maintained extremely significant superiority in BW and AC (*p* < 0.01), and significant superiority in BH, HH, BL, and IEW (*p* < 0.05). CG measurements remained comparable between groups (*p* > 0.05). At 30 months, polled cattle continued to show extremely significant advantages in BW, BL, and AC (*p* < 0.01). However, no significant differences were observed in BH, HH, CG, or IEW (*p* > 0.05) (Table 2).

### 3.2. Carcass Traits

Polled cattle exhibited extremely significantly higher carcass weight than horned cattle (*p* < 0.01). Additionally, both dressing percentage and meat yield were significantly greater in polled cattle compared to horned cattle (*p* < 0.05) (Table 3).

### 3.3. Meat Quality Characteristics

No significant differences (*p* > 0.05) were observed between polled and horned cattle in terms of muscle pH, color parameters (L*, a*, b*), cooking loss percentage, water-holding capacity, and shear force values (Table 4).

### 3.4. Detection of Candidate Genetic Markers (P_202ID_ and P_219ID_ Indels) for Polled Phenotype in Yanhuang Cattle

**Figure 1:**
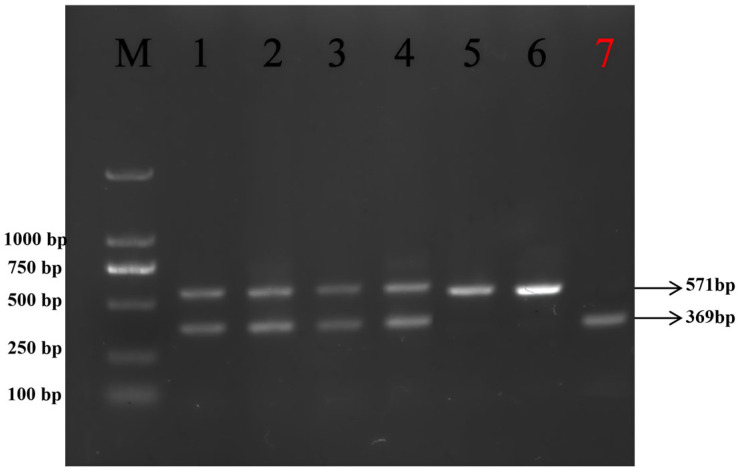
PCR amplification results of the P_202ID_ mutation site (DNA marker: MD114, Tian Gen). M denotes Marker. 1, 2, 3, 4, 5 and 6 are amplification products of polled cattle DNA (among them, lanes 1, 2, 3 and 4 are amplification products of P_C_/P_rs_, lanes 5 and 6 are amplification products of P_C_/P_C_); Lane 7 shows the amplified products of horned cattle (genotype P_rs_/P_rs_).

Genomic DNA extracted from Yanhuang cattle blood samples was amplified using the P_202ID_-specific primers. The PCR products were subsequently separated by 1% agarose gel electrophoresis. As shown in Figure 1, two distinct band patterns were observed: a 571 bp fragment indicating the presence of the P_202ID_ mutation, and a 369 bp fragment representing the wild-type allele.

The results showed that 7 polled cattle (3.5%) exhibited a homozygous P_C_/P_C_ genotype with only a 571 bp band, 93 polled cattle (46.5%) displayed a heterozygous P_C_/P_rs_ genotype with both 571 bp and 369 bp bands, and 100 horned cattle (50%) presented a homozygous P_rs_/P_rs_ genotype with only a 369 bp band (Table 5).

**Figure 2:**
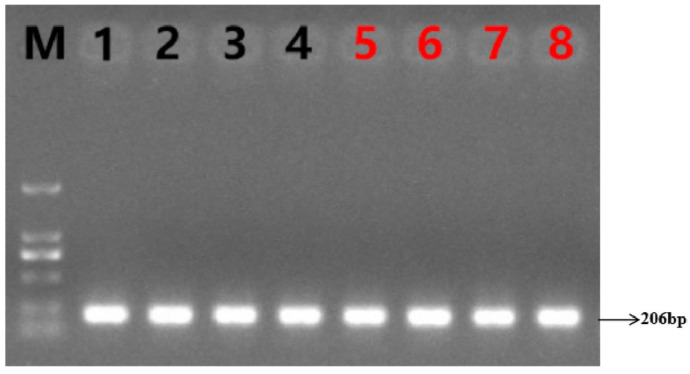
Results of P_219ID_ PCR amplification of the mutation site (DNA marker: MD114, Tian Gen). Lanes 1, 2, 3 and 4 are amplification products of polled yellow cattle DNA; lanes 5, 6, 7 and 8 represent the amplification products of angular yellow cattle.

Genomic DNA from Yanhuang cattle was amplified using P_219ID_-specific primers, followed by 1% agarose gel electrophoresis analysis. As shown in Figure 2, only the 206 bp fragment was observed.

### 3.5. Detection of Candidate Genetic Markers (P_80kbID_ and P_5ID_ Indels) for Polled Phenotype in Yanhuang Cattle

**Figure 3:**
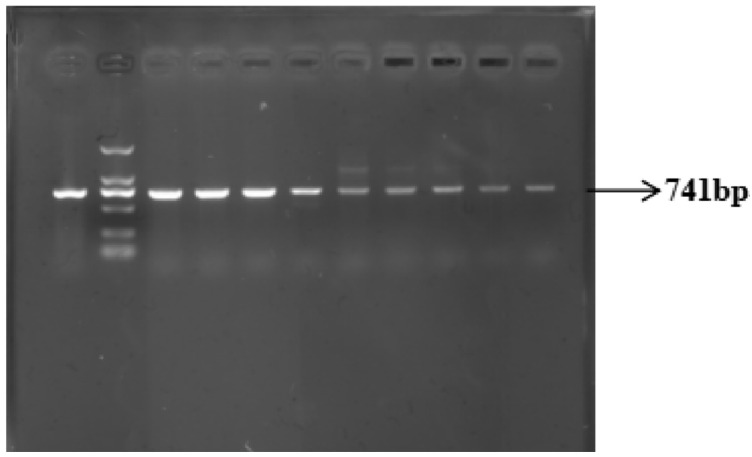
Results of P_80kbID_ PCR amplification of the mutation site (DNA marker: MD114, Tian Gen).

**Figure 4:**
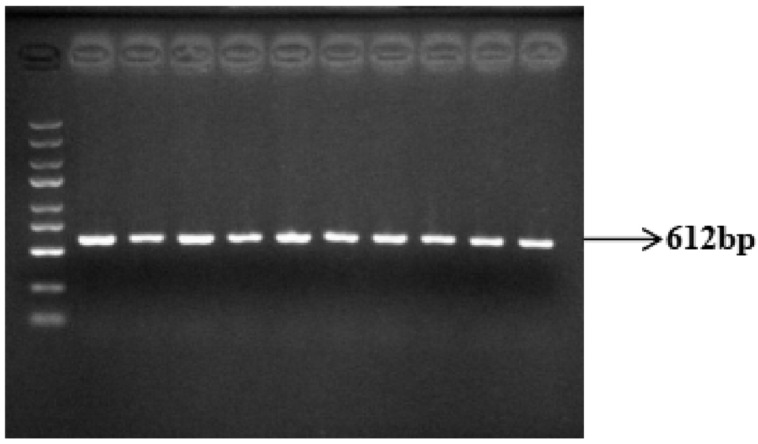
Results of P_5ID_ PCR amplification of the mutation site (DNA marker: D2000 plus DNA Ladder, Solarbio Life Sciences, Beijing, China).

Genomic DNA from Yanhuang cattle was amplified using P_80kbID_ and P_5ID_ specific primers, followed by agarose gel electrophoresis analysis. The PCR products showed expected fragment sizes of 741 bp for P_80kbID_ (Figure 3) and 612 bp for P_5ID_ (Figure 4), confirming successful amplification of both target regions. These specific and reproducible amplification products were deemed suitable for subsequent sequencing analysis.

**Figure 5:**
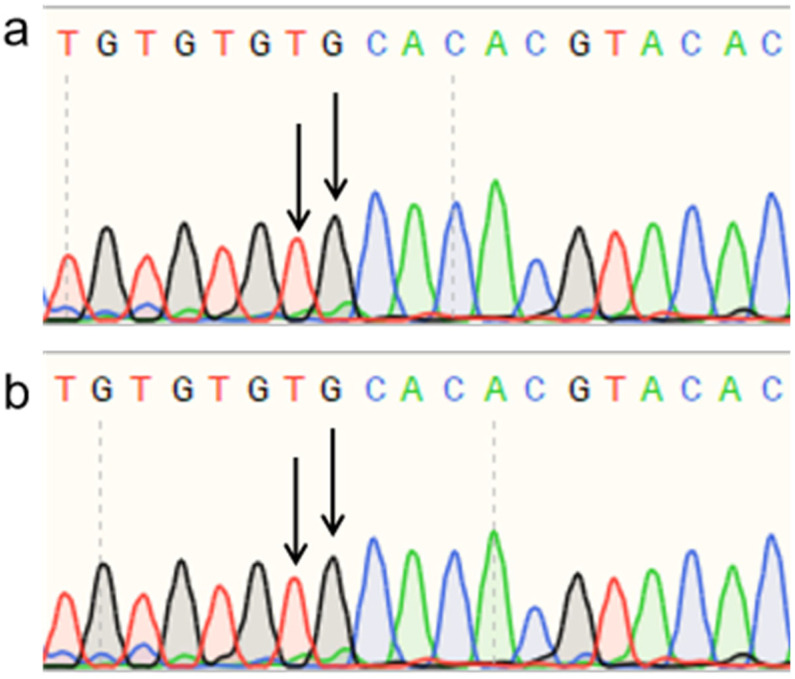
(**a**) Sequencing peak figures of the amplification product at the P_80kbID_ locus in polled Yanhuang cattle. (**b**) Sequencing peak figures of the amplification product at the P_80kbID_ locus in horned Yanhuang cattle.

**Figure 6:**
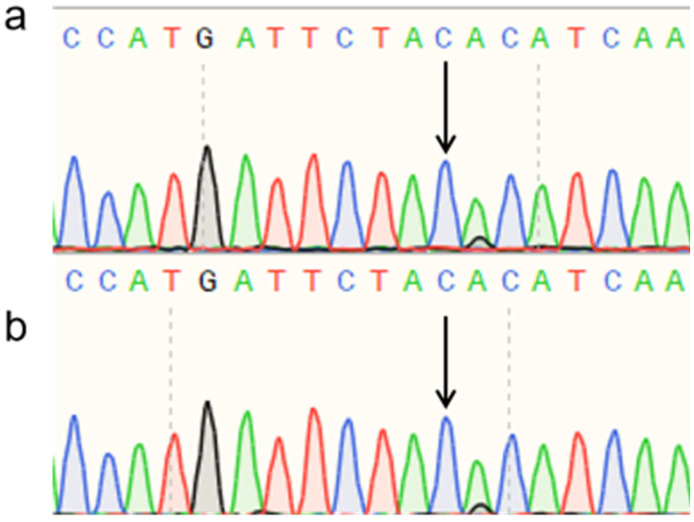
(**a**) Sequencing peak figures of the amplification product at the P_5ID_ locus in polled Yanhuang cattle. (**b**) Sequencing peak figures of the amplification product at the P_5ID_ locus in horned Yanhuang cattle.

Sanger sequencing of PCR products amplified from polled and horned Yanhuang cattle genomic DNA revealed identical sequences at both the P_80kbID_ (Figure 5) and P_5ID_ (Figure 6) loci. The sequencing chromatograms demonstrated complete sequence conservation between polled and horned individuals, indicating the absence of mutation at these loci in the Yanhuang cattle population. These results suggest that neither P_80kbID_ nor P_5ID_ represents a functional genetic marker for the polled phenotype in this breed.

### 3.6. Detection of Candidate Genetic Markers (P_G1855898A_, P_G1654405A_, and P_C1768587A_) for Polled Phenotype in Yanhuang Cattle

**Figure 7:**
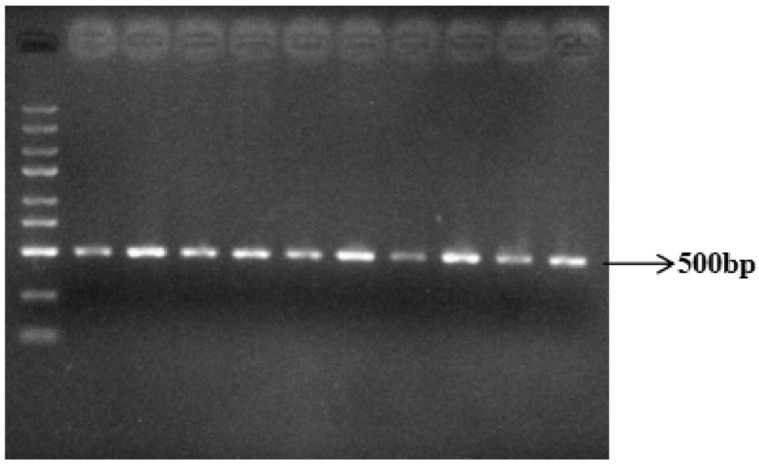
Results of P_G1855898A_ PCR amplification of the mutation site (DNA marker: D2000 plus DNA Ladder, Solarbio Life Sciences, Beijing, China).

**Figure 8:**
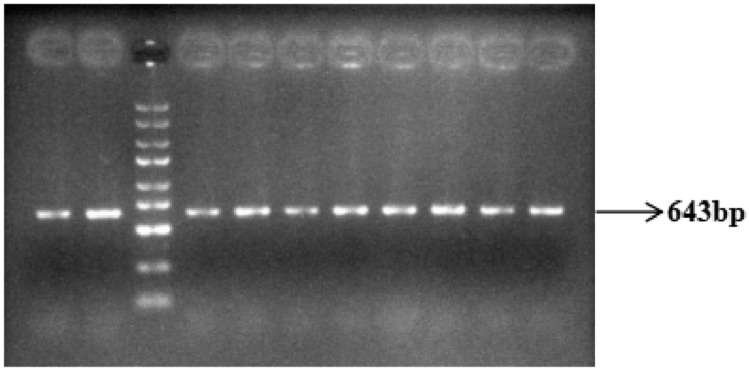
Results of P_G1654405A_ PCR amplification of the mutation site (DNA marker: D2000 plus DNA Ladder, Solarbio Life Sciences, Beijing, China).

**Figure 9:**
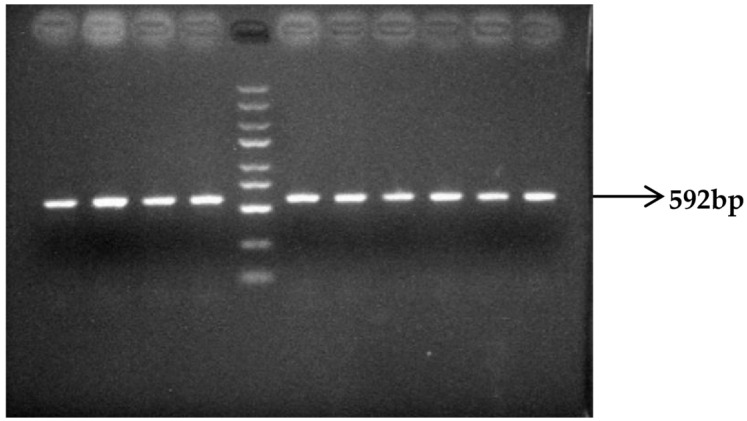
Results of P_C1768587A_ PCR amplification of the mutation site (DNA marker: D2000 plus DNA Ladder, Solarbio Life Sciences, Beijing, China).

DNA pools were constructed by combining equal amounts of genomic DNA from 50 polled and 50 horned Yanhuang cattle, respectively. These pooled DNA samples were amplified using three specific primer sets (P_G1855898A_, P_G1654405A_, and P_C1768587A_), followed by 1% agarose gel electrophoresis analysis. The results showed PCR products of expected sizes: 500 bp for P_G1855898A_ (Figure 7), 643 bp for P_G1654405A_ (Figure 8), and 592 bp for P_C1768587A_ (Figure 9). The specific and consistent amplification products confirmed the suitability of these markers for subsequent sequencing analysis.

**Figure 10:**
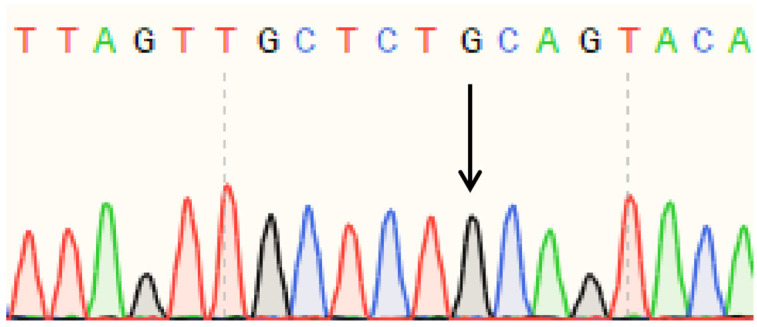
Sequencing peak figures of the Amplification Product at the P_G1855898A_ Locus in Yanhuang Cattle.

**Figure 11:**
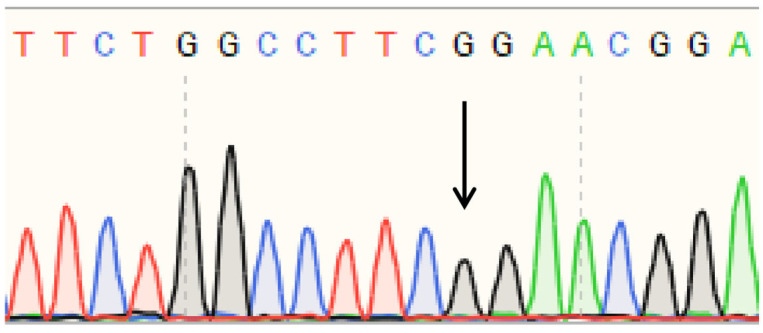
Sequencing peak figures of the Amplification Product at the P_G1654405A_ Locus in Yanhuang Cattle.

**Figure 12:**
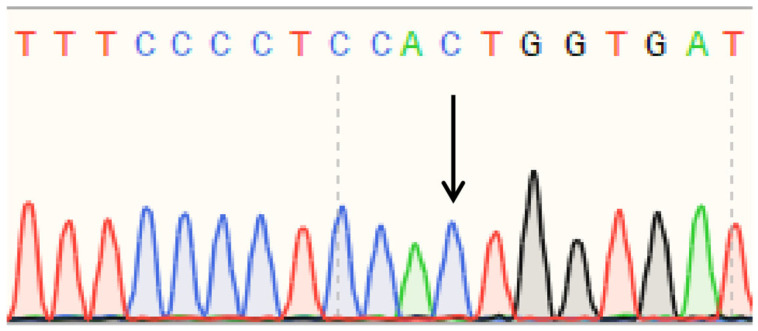
Sequencing peak figures of the Amplification Product at the P_C1768587A_ Locus in Yanhuang Cattle.

Sanger sequencing of PCR products amplified from the P_G1855898A_, P_G1654405A_, and P_C1768587A_ loci was performed (Figure 10, Figure 11 and Figure 12). The sequencing chromatograms revealed identical nucleotide sequences between polled and horned Yanhuang cattle at all three loci. These results demonstrate the absence of mutations at the P_G1855898A_, P_G1654405A_, and P_C1768587A_ loci in the studied Yanhuang cattle population, indicating these markers are not associated with the polled phenotype in this breed.

## 4. Discussion

### 4.1. Production Performance

Growth and development traits are the most easily measurable indicators for evaluating the economic characteristics of beef cattle. These traits exhibit moderate heritability, and their estimated breeding values (EBVs) are relatively accurate, making them key metrics for assessing beef cattle production performance [11]. The primary traits measured include body weight (weaning weight, yearling weight, 18-month weight, and 24-month weight) as well as body measurements such as BH, HH, BL, CG, AC and IEW.

The influence of horn traits on beef cattle body weight has not been reported, though related studies exist in sheep. Maerziya Yasen et al. [12] found that two-year-old hornless Kazakh sheep exhibited significantly higher body weight and wither height compared to horned Kazakh sheep. In this study, no significant difference was observed between hornless and horned Yanhuang cattle at six months of age. However, significant differences emerged at 12 months, and by 18 months onward, hornless Yanhuang cattle demonstrated markedly higher body weight than their horned counterparts, indicating superior growth performance. This disparity may arise because horned cattle divert partial energy and nutrients to horn growth and maintenance, whereas hornless cattle likely reallocate these resources to somatic growth and muscle development. Molecular studies reveal that horned fetuses develop distinct condensed cell clusters in the horn bud mesenchyme by day 58 of gestation, which may represent progenitor cells for horn formation—a process demanding substantial energy [13]. Additionally, the docility of hornless Yanhuang cattle might reduce energy expenditure from aggressive behaviors (e.g., fighting), further enhancing their growth rate and body weight.

Body height (BH) is a critical parameter for assessing growth performance in beef cattle and can facilitate breeding selection [14]. In this study, hornless Yanhuang cattle showed significantly greater BH than horned cattle at 12, 18 and 24 months of age, with the difference being highly significant at 24 months. No significant BH differences were detected at 6 or 30 months. This phenotypic variation may result from the hornless cattle’s reduced energy expenditure on agonistic behaviors, allowing greater nutrient allocation to growth. Furthermore, skeletal development predominantly occurs before 18 months, after which growth priorities transition from bone elongation to muscle accretion.

Hip height (HH) serves as a crucial parameter for evaluating beef cattle conformation and growth performance. Previous studies have demonstrated that HH at 8 months of age shows a strong correlation with final slaughter weight, indicating its predictive value for production performance [15]. However, the influence of horned/polled phenotypes on HH remains unexplored. In this study, hornless Yanhuang cattle exhibited significantly greater HH than horned cattle at 12, 18, and 24 months of age, with the difference being highly significant at 18 months. This phenomenon may be attributed to hornless cattle’s ability to redirect calcium, phosphorus and other resources from horn development to skeletal growth, particularly during rapid growth phases. The 18-month period represents a critical stage for skeletal development, when such resource allocation differences become most pronounced.

Body length (BL), another essential indicator for assessing early growth patterns in cattle [16], reflects longitudinal skeletal and muscular development [17]. Our results demonstrated that hornless Yanhuang cattle had significantly greater BL than horned cattle at 6, 18, 24, and 30 months, with the difference reaching high significance at 30 months. Although hornless cattle showed numerically greater BL at 12 months, the difference was not statistically significant. This progressive advantage may result from continuous energy reallocation in hornless cattle, as the absence of horn tissue development permits greater nutrient investment in body growth. The cumulative effect of this long-term energy redistribution likely explains the increasingly significant differences observed with advancing age.

Chest girth (CG), as a key morphometric parameter, indicates thoracic development and demonstrates associations with cardiopulmonary capacity and muscularity [18]. Zhang Zhifei [19] reported no significant CG differences between one-year-old hornless and horned yaks. Our results similarly showed no significant CG variations between hornless and horned Yanhuang cattle at 6, 12, 24, or 30 months of age, confirming Zhang’s findings.

Abdominal girth (AG) represents an important health and productivity indicator, reflecting rumen volume, digestive function, fat deposition, and muscle development [20]. No previous studies have examined horned/hornless phenotype effects on AG. In this study, hornless Yanhuang cattle displayed significantly larger AG than their horned counterparts at 18, 24, and 30 months, with no significant differences at other ages. This likely results from the 18-month developmental transition from skeletal to muscular growth phases, where the energy conservation advantage becomes pronounced in abdominal development.

Ischial end width (IEW), a crucial skeletal development parameter, reflects beef cattle’s frame size potential and muscle attachment capacity [21]. No previous studies have reported horned/hornless phenotype effects on IEW. Our results revealed that hornless Yanhuang cattle had significantly greater IEW than horned cattle at 6, 12, and 24 months of age, with highly significant differences observed at 6 and 12 months. While hornless cattle maintained numerically higher IEW values at 18 and 30 months, these differences were not statistically significant. These findings suggest that genetic influences on IEW are most pronounced during rapid skeletal growth phases. The hornless phenotype appears to confer skeletal growth advantages during critical developmental windows, resulting in significant dimensional differences. The attenuation of these differences post-18 months likely reflects the natural plateauing of skeletal growth. Further research is needed to elucidate the precise mechanisms underlying these phenotypic variations.

### 4.2. Molecular Identification of Candidate Genetic Markers

Horns represent a significant species-specific characteristic in cattle and serve as their primary defensive tool in natural environments [22]. Under natural conditions, horns provide protection against predators and facilitate competition for food resources [23]. However, with the transition from draft to beef production systems, the presence of horns in intensively managed cattle has become problematic, leading to increased aggression, human injuries, and transportation challenges [24].

Conventional dehorning practices are not only labor-intensive and costly [25], but also raise serious animal welfare concerns due to the substantial pain inflicted [26]. Maci L. Mueller et al. demonstrated through 20-year breeding simulations that traditional selection methods yield slow genetic gains, while incorporating gene editing significantly accelerates genetic improvement rates [27]. Consequently, molecular breeding techniques for developing polled cattle have gained increasing research attention.

The P_202ID_ mutation was initially identified in several European beef breeds of Celtic origin. This complex indel variant involves a 212 bp sequence duplication (1,705,834–1,706,045 bp) replacing a 10 bp segment (1,706,051–1,706,060 bp) on chromosome 1 [6]. However, Sadie L. Hennig et al. [28] reported that CRISPR-Cas9-mediated deletion of the polled locus (including a 133 bp region containing the 10 bp sequence) failed to completely suppress horn bud development in embryos carrying biallelic deletions, suggesting the 10 bp deletion alone cannot induce the polled phenotype.

In this study, we detected the P_202ID_ mutation in all 100 polled Yanhuang cattle (93 heterozygous and 7 homozygous), while none of the 100 horned individuals carried this variant. These findings are consistent with Hao Qinqin et al.’s [29] observations in Simmental cattle, where 108 polled animals uniformly possessed the P_202ID_ mutation.

Medugorac et al. [8] identified a horn-related quantitative trait locus (QTL) on chromosome 1 in Mongolian yaks, characterized by a 219 bp duplicated insertion located 61 bp downstream of the original sequence (starting at position 1,976,128 bp). Chen et al. [30] subsequently detected this P_219ID_ mutation in polled Sichuan cattle. In contrast to these findings, our genotyping analysis of 100 polled Yanhuang cattle revealed no individuals carrying the P_219ID_ mutation. The observed phenotypic differences are primarily attributable to breed-specific genetic architectures.

The P_80kbID_ variant, characterized by an 80 kb duplication (1,909,352–1,989,480 bp) accompanied by a 2 bp deletion on bovine chromosome 1 [6], primarily regulates horn phenotypes in dairy cattle breeds. This mutation predominates in Holstein populations from the Netherlands and Germany, while both P_80kbID_ and P_202ID_ variants coexist in French and Swiss cattle populations. Chen et al. [30] identified the P_80kbID_ mutation in Sichuan cattle through genotyping 48 polled and 16 horned individuals. In this study, we developed specific primers targeting the 2 bp deletion within P_80kbID_ for Sanger sequencing analysis. Our results demonstrated the complete absence of the P_80kbID_ mutation in all 100 polled Yanhuang cattle examined.

Regarding the P_5ID_ variant, this Holstein-specific polymorphism involves a 12 bp replacement (ttctcagaatag) of a 7 bp sequence (cgcatca; RefSeq coordinates: 1,649,163–1,649,169) at 1.648–2.027 Mb on chromosome 1, resulting in a net 5 bp insertion [6]. Genotyping focused on detecting sequence alterations at position 1,649,163. Through PCR amplification and sequencing, we confirmed the absence of the P_5ID_ mutation in all 100 polled Yanhuang cattle, as well as in horned controls.

P_G1855898A_, P_C1768587A_, and P_G1654405A_ were identified on bovine chromosome 1. Specifically, P_G1855898A_ refers to a G-to-A substitution at position 1,855,898, P_C1768587A_ denotes a C-to-A mutation at position 1,768,587, and P_G1654405A_ indicates a G-to-A transition at position 1,654,405 [31]. Since these are all single-nucleotide polymorphisms (SNPs), this study employed a random sampling-based pooling strategy for Sanger sequencing. The results confirmed the absence of these three polled mutation sites in the hornless Yanhuang cattle population.

## 5. Conclusions

In summary, this study confirms a significant association between the polled trait and growth performance in Yanyuang cattle. Molecular identification indicates that the P202ID mutation can serve as a reliable molecular marker for polled breeding in Yanyuang cattle, providing theoretical and practical support for the development of new superior polled lines.

However, the molecular mechanism underlying the effect of the polled trait on production performance in Yanyuang cattle has not been thoroughly investigated in this study. Future research may optimize the sample size and experimental conditions to improve the universality and reliability of the results.

## Figures and Tables

**Table 1:** Primer information of candidate genetic marker loci for polled phenotype.

Candidate Mutations	Primer Sequences (5′-3′)	Binding Sites of Primer	Annealing Temperature
P_202ID_	F:TCAAGAAGGCGGCACTATCT R:TGATAAACTGACCCTCTGCCTATA	Chr1:1,705,792–1,705,811	59 °C
		Chr1:1,706,160–1,706,137	
P_80kbID_	F: GGTGAACAGATGAGGTCAGAGA R: AGGCAGGAGAAGGTTACAAGAA	Chr1:1,909,358–1,909,377	59 °C
		Chr1:1,909,509–1,909,490	
P_5ID_	F:CATCGGCTCTTCATACAATACAATC R:ACGCTATAGTCAGAGATCACAAAGA	Chr1:1,648,889–1,648,913	59 °C
		Chr1:1,649,500–1,649,476	
P_G1855898A_	F:TCCAGCAATGAGACAAAATG R:TCCATCTAATCTCCCCCTGTT	Chr1:1,855,617–1,855,636	55 °C
		Chr1:1,856,116–1,856,096	
P_C1768587A_	F:AGGAGGTTGGCATTTGATTG R:AAATCCAGAGTTGAGCCGAT	Chr1:1,768,264–1,768,283	58 °C
		Chr1:1,768,855–1,768,836	
P_G1654405A_	F:GCAATAACAAAACCGAAAGCAGGAT R:CTGAACTTACAGGTGAAGGAATCTG	Chr1:1,654,055–1,654,079	60 °C
		Chr1:1,654,697–1,654,673	
P_219ID_	F:TGAAACTTTTGGCAAGCATGA R:CTGTGTCTCCTCGTGAGTCC	Chr1:1,976,113–1,976,133	59 °C
		Chr1:1,976,318–1,976,299	

**Table 2:** Comparison of Growth Traits in Yanhuang Cattle at Different Months of Age.

Month of Age	Traits	Polled Yanhuang Cattle	Horned Yanhuang Cattle
6	BW (kg)	152.28 ± 24.97	142.43 ± 13.51
	BH (cm)	100.38 ± 5.56	98.13 ± 4.92
	HH (cm)	106.02 ± 5.45	103.30 ± 3.61
	BL (cm)	103.63 ± 6.36 ^a^	100.07 ± 4.73 ^b^
	CG (cm)	125.00 ± 6.37	123.77 ± 6.63
	AC (cm)	145.97 ± 9.90	141.63 ± 10.88
	IEW (cm)	9.98 ± 1.25 ^A^	8.90 ± 1.12 ^B^
12	BW (kg)	225.30 ± 19.23 ^a^	213.88 ± 19.37 ^b^
	BH (cm)	106.55 ± 5.22 ^a^	103.70 ± 3.37 ^b^
	HH (cm)	113.07 ± 4.31 ^a^	110.30 ± 3.50 ^b^
	BL (cm)	114.37 ± 6.10	112.60 ± 6.72
	CG (cm)	144.17 ± 9.30	140.77 ± 7.56
	AC (cm)	175.30 ± 11.71	175.27 ± 9.12
	IEW (cm)	9.85 ± 2.52 ^A^	8.03 ± 2.24 ^B^
18	BW (kg)	363.17 ± 18.51 ^A^	319.18 ± 16.77 ^B^
	BH (cm)	124.13 ± 5.62 ^A^	119.57 ± 4.80 ^B^
	HH (cm)	129.67 ± 5.82 ^A^	124.63 ± 5.34 ^B^
	BL (cm)	138.90 ± 6.95 ^a^	135.43 ± 5.93 ^b^
	CG (cm)	170.93 ± 6.65 ^a^	165.67 ± 8.57 ^b^
	AC (cm)	203.20 ± 9.39 ^A^	196.30 ± 8.97 ^B^
	IEW (cm)	13.90 ± 2.45	13.07 ± 3.22
24	BW (kg)	483.15 ± 29.22 ^A^	421.08 ± 26.36 ^B^
	BH (cm)	129.57 ± 3.70 ^a^	126.97 ± 4.30 ^b^
	HH (cm)	135.30 ± 2.97 ^a^	133.17 ± 4.03 ^b^
	BL (cm)	150.07 ± 6.76 ^a^	144.43 ± 9.98 ^b^
	CG (cm)	186.83 ± 8.03	181.87 ± 12.85
	AC (cm)	229.77 ± 11.20 ^A^	220.13 ± 11.84 ^B^
	IEW (cm)	13.50 ± 2.37 ^a^	14.90 ± 2.71 ^b^
30	BW (kg)	608.17 ± 28.16 ^A^	548.95 ± 24.38 ^B^
	BH (cm)	134.92 ± 5.04	132.80 ± 4.22
	HH (cm)	139.48 ± 4.57	137.45 ± 4.40
	BL (cm)	158.03 ± 10.61 ^A^	154.43 ± 6.82 ^B^
	CG (cm)	201.93 ± 6.20	201.93 ± 10.34
	AC (cm)	248.97 ± 8.01 ^A^	236.10 ± 12.18 ^B^
	IEW (cm)	13.53 ± 2.22	14.28 ± 2.47

Note: The same lowercase letter or no letter in the same shoulder label means no significant difference (*p* > 0.05), different lowercase letters indicate a significant difference (*p* < 0.05), and different uppercase letters indicate a very significant difference (*p* < 0.01).

**Table 3:** Comparison of carcass traits between Polled and horned Yanhuang cattle.

Traits	Polled Yanhuang Cattle	Horned Yanhuang Cattle
Carcass weight (kg)	340.74 ± 25.48 ^A^	296.95 ± 20.35 ^B^
Dressing percentage (%)	56.09 ± 4.32 ^a^	54.07 ± 2.55 ^b^
Net meat percentage (%)	49.08 ± 1.82 ^a^	48.15 ± 1.62 ^b^

Note: The same lowercase letter or no letter in the same shoulder label means no significant difference (*p* > 0.05), different lowercase letters indicate a significant difference (*p* < 0.05), and different uppercase letters indicate a very significant difference (*p* < 0.01).

**Table 4:** Comparison of meat quality traits between polled and horned Yanhuang cattle.

Traits	Polled Yanhuang Cattle	Horned Yanhuang Cattle
pH	5.69 ± 0.24	5.70 ± 0.36
L*	32.72 ± 2.77	32.56 ± 2.31
a*	18.30 ± 2.80	18.19 ± 2.87
b*	13.83 ± 2.08	13.90 ± 2.09
Cooking loss (%)	28.47 ± 1.27	28.72 ± 2.53
Water-holding capacity	43.73 ± 2.61	43.33 ± 3.47
Shear force (N)	98.31 ± 8.94	98.41 ± 7.43

**Table 5:** PCR amplification fragment size, genotype and genotype frequency of the mutation site P_202ID_ of Yanhuang cattle.

Items	Sample Numbers	Fragment Length	Genotype	Genotype Frequency
Polled Yanhuang cattle	100	369 bp/571 bp (93)	P_c_/P_rs_	46.5%
		571 bp (7)	P_c_/P_c_	3.5%
Horned Yanhuang cattle	100	369 bp (100)	P_rs_/P_rs_	50%

## Data Availability

The data presented in this study are available on request from the corresponding authors.
